# Solid Dispersion Characteristics of an Oscillatory Microfluidic Mixer: An Experimental and Numerical Investigation

**DOI:** 10.3390/mi17091036

**Published:** 2026-08-29

**Authors:** Yao Lu, Haixuan Sun, Yifan Qin

**Affiliations:** 1 Suzhou Institute of Biomedical Engineering and Technology, Chinese Academy of Sciences, Suzhou 215163, China; luyao@sibet.ac.cn; 2 School of Biomedical Engineering (Suzhou), University of Science and Technology of China, Hefei 230026, China

**Keywords:** oscillatory flow, solid-liquid mixing, microfluidics, coagulation assay

## Abstract

Point-of-care (POC) diagnostic testing based on microfluidic technology plays an important role in coagulation management. Its precision is, however, limited by the inefficient mixing between blood and solid activators in laminar microfluidic flow. In this study, a novel micromixer incorporating periodic oscillatory flow was developed to enhance blood-activator mixing. A computational fluid dynamic (CFD) model was established to investigate the microscale hydrodynamic characteristics and solid dispersion behavior in the oscillatory multiphase flow system. The numerical predictions were validated against tracer mixing experiments. Sample calculations for the air-liquid-solid flow system demonstrated that the initial loading position of activator particles significantly affected the dispersion efficiency due to the spatial variation in the radial velocity field. Compared with the center-initialized case, the solid dispersion level in the corner-initialized case decreased by approximately 42% after two oscillation cycles. Furthermore, the influence mechanism of oscillation period on solid dispersion was clarified through multi-physics coupling analysis. This study provided new insights into solid–liquid mixing in oscillatory microfluidic systems and established an effective CFD-based framework for optimizing microdevice design and operating conditions.

## 1. Introduction

**Figure 1:**
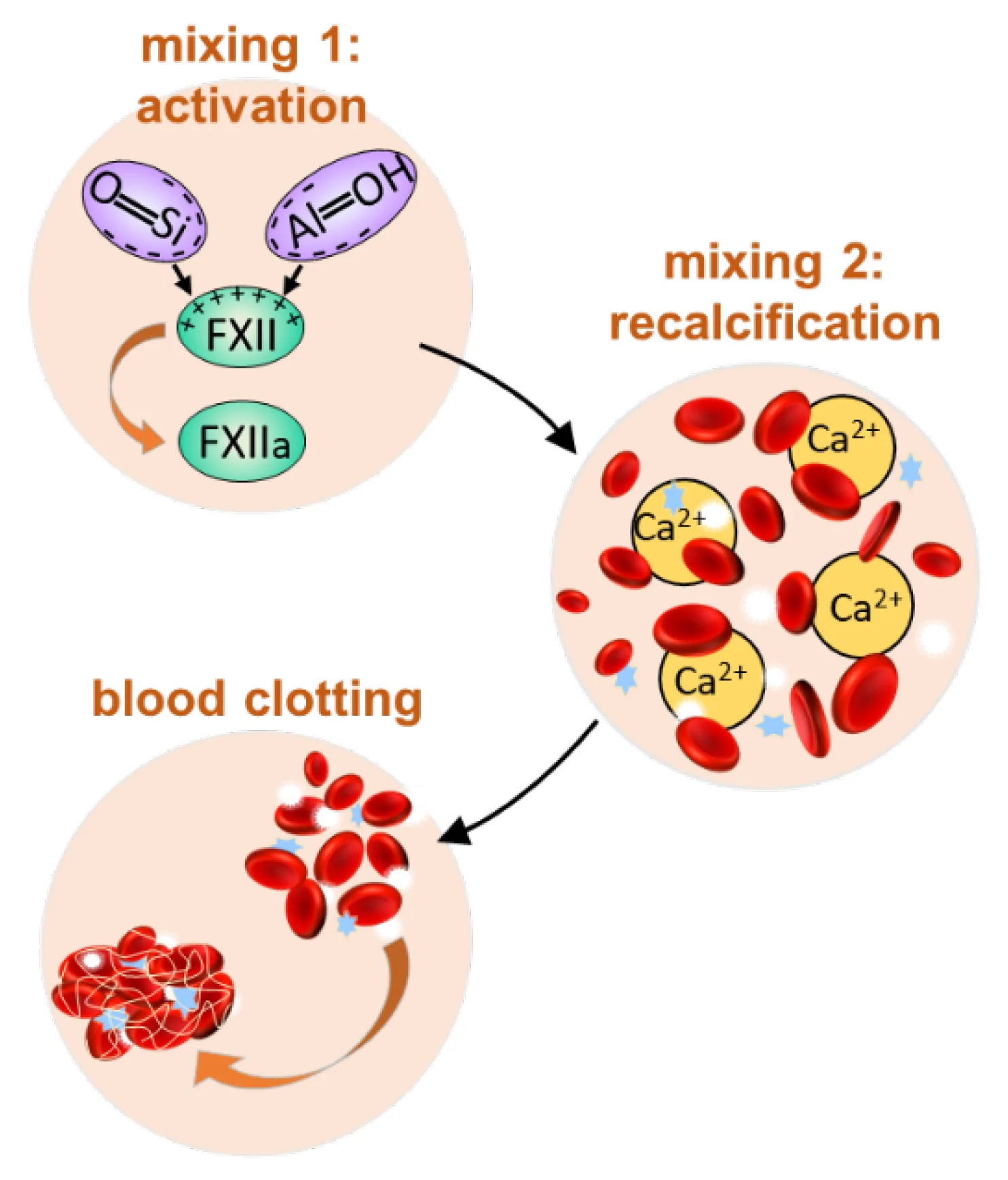
Schematic diagram of kaolin-based coagulation cascade.

Microfluidic technology has revolutionized point-of-care testing by enabling the precise manipulation of fluids at the sub-millimeter scale [1]. Its advantages, including low reagent consumption, rapid analysis, and high integration, make it an ideal platform for biochemical assays [2,3]. However, the miniaturization of fluidic channels introduces a fundamental hydrodynamic challenge, that is the dominance of laminar flow. In microchannels, the Reynolds number (Re) is typically very low, indicating that viscous forces significantly outweigh inertial forces. Consequently, the mixing of reagents and samples is governed by diffusion of the media, which is an inherently slow process [4,5]. This mixing limitation is particularly critical in coagulation assays, especially for the activated clotting time (ACT). As shown in Figure 1, the coagulation cascade is initiated when Factor XII in whole blood comes into contact with negatively charged particles. Unlike homogeneous reactions involving only soluble reagents, this process constitutes a heterogeneous solid–liquid reaction system [6]. It requires the rapid and uniform distribution of activator particles throughout the blood to trigger the intrinsic coagulation pathway as synchronously as possible. Under laminar flow conditions in microchannels, solid activators, such as kaolin particles or glass beads, are prone to sedimentation due to gravity and the absence of flow-induced disturbances. Inefficient mixing causes the activator particles to remain aggregated or settle at the bottom of the channel, leading to a false prolongation of the clotting time [7]. Therefore, achieving rapid and homogeneous mixing in a solid–liquid microfluidic system is not merely an optimization objective but a prerequisite for accurate diagnosis.

To overcome the limitations of diffusion-dominated laminar flow, various micro-mixing strategies have been developed over the past several decades. These strategies can generally be classified into active and passive approaches based on whether external energy is applied to facilitate fluid mixing [8]. Passive micromixers rely on complex channel geometry design, e.g., curved microchannels [9], gear-shaped microchannels [10], sharp corners [11], and two-layer structures [12], to generate chaotic advection and enhance fluid contact. Although they are easy to fabricate and readily integrated into microfluidic devices, passive micromixers generally exhibit limited mixing efficiency, particularly in heterogeneous systems containing dense particles. In contrast, active micromixers employ external energy sources, such as acoustic [13], magnetic [14] or thermal fields [15], to perturb laminar flow more efficiently. However, these active control technologies require complex external peripherals, reducing their suitability for compact and portable POC devices. Therefore, a simple, highly efficient mixing strategy is required to achieve rapid and homogeneous solid–liquid mixing for ACT assays.

Oscillatory flow has emerged as a promising hydrodynamic approach for enhancing mixing in reactors [16]. By imposing a periodic reciprocating motion on the fluid, oscillatory flow generates secondary flow vortices and continuously stretches fluid interfaces, thereby promoting efficient mixing even in straight channels. More importantly, oscillation-driven mixing is largely independent of the net flow rate. Consequently, it enables effective mixing under low flow rate conditions, where conventional mixing strategies often exhibit poor hydrodynamic performance. This capability allows the reactor volume to be reduced, facilitating the development of compact systems [17]. However, the mechanisms by which oscillatory flow influences the mixing of whole blood and solid activators in microchannels remain poorly understood.

This study developed a novel microfluidic mixer that generates reciprocating oscillatory flow using an integrated finger-actuated unit. Although the liquid–liquid mixing performance of the micromixer can be readily characterized in virtue of visual tracer techniques, it is challenging to evaluate the dispersion of activator particles in a liquid via experimental methods. Computational fluid dynamic, which has attracted increasing attention in the field of microfluidics [18], was employed to investigate the microscale hydrodynamic characteristics and solid dispersion behavior of three-phase oscillatory flow. After validating the model through a tracer mixing experiment, CFD-aided parametric analysis was performed to identify the optimal operating parameters for blood–activator mixing. The ultimate aim of this work was to provide a robust mixing strategy that eliminates the false prolongation of ACT assays caused by inadequate mixing.

## 2. Materials and Methods

### 2.1. Geometry and Operation of the Micromixer

**Figure 2:**
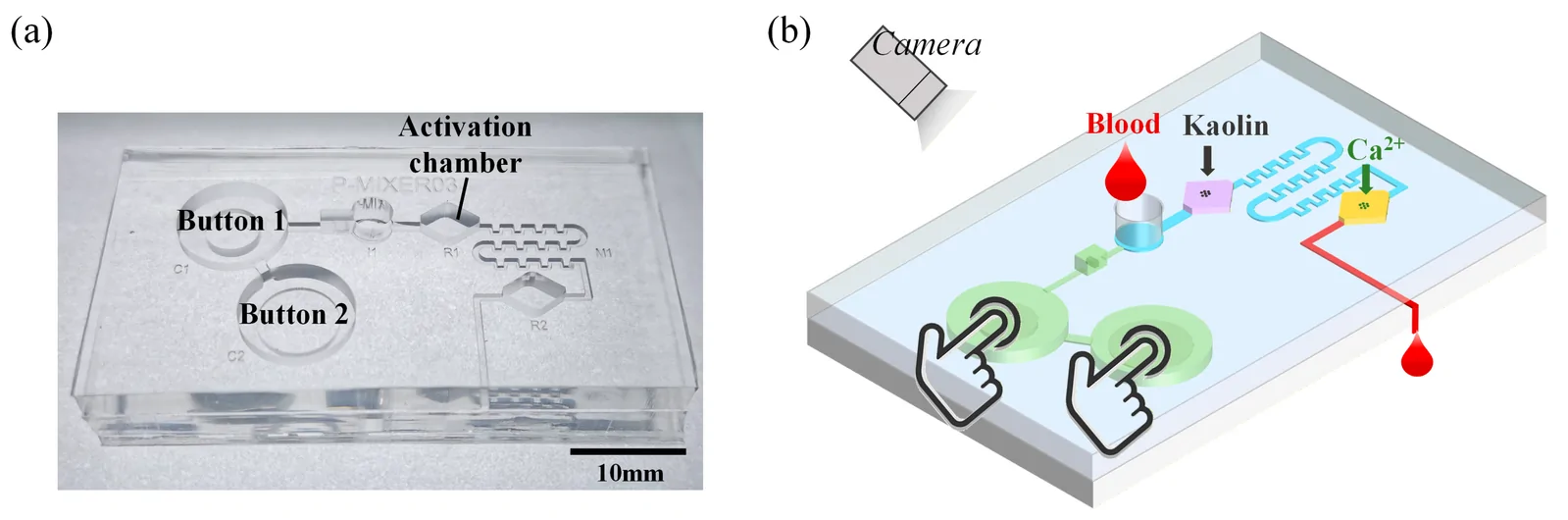
(**a**) The microfluidic mixer for coagulation activation. (**b**) Schematic diagram of testing setups.

As shown in Figure 2a, a microfluidic chip fabricated from PDMS has overall dimensions of 50 mm × 28 mm × 3 mm. It consists of two pneumatic actuation buttons, a blood chamber, an activation chamber, a microfluidic channel and a recalcification chamber. The air-filled, finger-actuated buttons were designed to perform different operational steps, as illustrated in Figure 2b. When the air in the first button (approximately 48 μL) is fully discharged, the blood sample in the cylindrical chamber can be driven through the activation chamber containing kaolin and reach the end of the microchannel. The second button, with a maximum air volume of approximately 70 μL, provides sufficient pressure to transport the activated blood into the recalcification chamber where Ca^2+^ is added. Simultaneous actuation of both buttons expels all of the liquid from the chip. Repeated pressing and releasing of the buttons generates periodic pneumatic pressure, driving the fluid back and forth within confined regions to produce reciprocating oscillatory flow and promote mixing with the corresponding reagents.

### 2.2. Materials and Implementation of Tracer Mixing Experiment

The mixing performance of the microfluidic device was characterized using a 20% glycerol aqueous solution (density: 1050 kg/m^3^) as the working medium because its physical properties are similar to those of whole blood. The dynamic viscosity of the solution was measured to be 2.6 × 10^−3^ Pa·s using a rotational rheometer. A 0.5% Allura Red solution, prepared by dissolving Allura Red (Shanghai Aladdin Co., Ltd., Shanghai, China) in 20% glycerol aqueous solution, was used as the tracer. The experimental procedure was as follows. After the high-definition camera was positioned for recording, 30 μL of transparent 20% glycerol aqueous solution was introduced into the activation chamber, while 5 μL of the deep-red 0.5% Allura Red solution was introduced into the blood chamber. The first pneumatic button was then repeatedly actuated to drive the two solutions into reciprocating flow between the activation chamber and the microchannel, thereby promoting rapid mixing. The experiment was terminated when the fluid throughout the device exhibited a uniform color, indicating complete mixing.

Following the image analysis method presented by Xiao et al. [19], the mixing level within the activation chamber was defined as a quantitative criterion for evaluating the mixing performance of the microfluidic device experimentally. Color images of the specified fluid domain at different time points were extracted from the recorded video and converted into RGB triplets, triplets ($R_{xy}\left(t\right),G_{xy}\left(t\right),B_{xy}\left(t\right)$), using MATLAB 2024a. The subscripts *x* and *y* denote the row and column indices of each pixel in the image, respectively. The color deviation between the sample at a given time and the fully mixed reference state was calculated using the following equation:

(1) $$d_{xy}\left(t\right)=\sqrt{{\left(R_{xy}\left(t\right)-R_{xy}\left(\infty \right)\right)}^{2}+{\left(G_{xy}\left(t\right)-G_{xy}\left(\infty \right)\right)}^{2}+{\left(B_{xy}\left(t\right)-B_{xy}\left(\infty \right)\right)}^{2}}$$

where the triplet ($R_{xy}\left(\infty \right),G_{xy}\left(\infty \right),B_{xy}\left(\infty \right)$) represents the pixel values of a sample approaching the fully mixed state with nearly 100% homogeneity.

A normalized mixing level (dimensionless) at a specific time point was then determined based on the average color deviation over the entire fluid domain:

(2) $$M_{exp}\left(t\right)=\frac{\bar{d_{xy}}\left(0\right)-\bar{d_{xy}}\left(t\right)}{\bar{d_{xy}}\left(0\right)}\times 100\%$$

where $\bar{d_{xy}}\left(0\right)$ represents the average color deviation at the initial state. According to Equation (2), the mixing level is 0% at the initial state and approaches 100% when complete mixing is achieved.

## 3. Computational Fluid Dynamics

In this context, a three-dimensional (3D) multiphysics CFD model was constructed to resolve two multiphase flow systems. The first gas–liquid flow system was established to reproduce the mixing process of the glycerol aqueous solution and the Allura Red tracer in the experimental validation. The second system is a gas–liquid–solid flow model developed to investigate the dispersion behavior of solid activator particles in blood, a process that is difficult to directly observe and quantify using experimental approaches. By incorporating particle dynamics into the multiphase framework, this model provides insights into the interaction between oscillatory flow and solid transport.

### 3.1. Governing Equations

The conservation equations of the multiphase flow system were solved using the Eulerian multiphase model, considering the relatively high proportion of solid phase in a given region. In microchannels, the flow was typically considered laminar. All the phases involved in this study were treated as incompressible fluids. The continuity and momentum equations for each phase can be written as the following general forms, respectively:

(3) $$\frac{\partial }{\partial t}\left(\alpha_{q}\rho_{q}\right)+\nabla \cdot \left(\alpha_{q}\rho_{q}{\vec{v}}_{q}\right)=0$$

(4) $$\frac{\partial }{\partial t}\left(\alpha_{q}\rho_{q}{\vec{v}}_{q}\right)+\nabla \cdot \left(\alpha_{q}\rho_{q}{\vec{v}}_{q}{\vec{v}}_{q}\right)=\alpha_{q}\nabla p-\nabla p_{s}+\nabla \cdot {\overset{̿}{\tau }}_{q}+\alpha_{q}\rho_{q}\vec{g}+K\left({\vec{v}}_{p}-{\vec{v}}_{q}\right)+{\overset{⃑}{F}}_{s}$$

where $\alpha_{q}$ [-], $\rho_{q}$ [kg/m^3^] and ${\vec{v}}_{q}$ [m/s] denote the volume fraction, density, and velocity of a phase, respectively. p [Pa] is the pressure shared by all three phases. The vector $\vec{g}$ [m/s^2^] is the gravitational acceleration. The term $p_{s}$ denotes the solid pressure, which is considered only when solving the momentum conservation equation of the solid phase. The stress–strain tensor of each phase ${\overset{̿}{\tau }}_{q}$ [Pa], follows the constitutive relationship for incompressible fluids, where the contribution of volumetric expansion is neglected.

(5) $${\overset{̿}{\tau }}_{q}=\alpha_{q}\mu_{q}\nabla {\vec{v}}_{q}$$

The interphase momentum exchange coefficient, (K) [kg/(m^3^·s)], was determined using different drag models for fluid–fluid and fluid–solid interactions. For fluid–fluid flows, the drag coefficient was calculated using the Schiller–Naumann model [20], whereas the Wen–Yu model [21] was employed for fluid–solid flows. Accordingly, the interphase momentum exchange coefficient can be expressed as

(6) $$K_{pq}=\{\begin{array}{c} \begin{array}{cc} \frac{18\mu_{q}\alpha_{q}(1+0.15(\frac{p_{q}\left|{\vec{v}}_{p}-{\vec{v}}_{q}\right|}{\mu_{q}}{)}^{0.687})}{d_{p}^{2}} & for\;fluid\text{–}fluid\;flows \end{array}\\ \begin{array}{cc} \frac{3\alpha_{s}\alpha_{q}p_{q}\left|{\vec{v}}_{p}-{\vec{v}}_{q}\right|}{4v_{r}^{2}d_{p}^{2}}(0.63+\sqrt{\frac{23.04\mu_{q}v_{r}}{p_{q}\left|{\vec{v}}_{p}-{\vec{v}}_{q}\right|}}{)}^{2} & for\;fluid\text{–}solid\;flows \end{array} \end{array}$$

where $d_{p}$ [m] is the diameter of the solid particles. $\mu_{q}$ is the dynamic viscosity of a phase. $v_{r}$ [m/s] denotes the terminal velocity correlation for the dispersed solid phase. The surface tension between the liquid and gas phases was modeled using the continuum surface force (CSF) model proposed by Brackbill et al. [22], which converts the interfacial surface tension into an equivalent volumetric force acting within the interface region. The surface tension force is expressed as

(7) $${\overset{⃑}{F}}_{s}=\frac{2p_{q}\kappa_{q}\sigma_{pq}\nabla \alpha_{q}}{p_{p}+p_{q}}$$

where $\sigma_{pq}$ denotes the surface tension coefficient between phases *p* and *q*. The interface curvature $\kappa_{q}$ is defined as the divergence of the unit normal vector to the interface.

To quantify the liquid–liquid mixing process, the transport of the tracer was described by the species transport equations:

(8) $$\frac{\partial }{\partial t}\left(\alpha_{q}\rho_{q}Y_{i}\right)+\nabla \cdot \left(\alpha_{q}\rho_{q}{\vec{v}}_{q}Y_{i}\right)=-\nabla \cdot \alpha_{q}{\overset{⃑}{J}}_{i}$$

(9) $${\overset{⃑}{J}}_{i}=-\rho_{q}D_{i}\nabla Y_{i}$$

where $Y_{i}$ [-] and ${\overset{⃑}{J}}_{i}$ [kg/m^2^·s] denote the local mass fraction and diffusion flux of species *i* in the mixture, respectively.

Since the tracer concentration varies spatially and temporally during the mixing process, the local density of the liquid phase was calculated according to the volume-weighted mixing law:

(10) $$\rho_{q}=\sum_{i}\rho_{i}y_{i}$$

where $\rho_{i}$ [kg/m^3^] and $y_{i}$ [-] are the density and volume ratio of component *i*, respectively.

According to the Wilke-Chang equation [23], the diffusion coefficient is inversely proportional to the fluid viscosity. Based on the experimentally measured diffusion coefficient in water [24], the diffusion coefficient in the 20% glycerol aqueous solution was estimated to be 2.1 × 10^−10^ m/s^2^. When simulating the dispersion of solid activator particles under oscillatory flow, the liquid phase was specified as blood. Owing to the relatively low shear rates within the activation chamber, blood exhibits pronounced non-Newtonian behavior. Accordingly, the Carreau model [25] was adopted to describe the shear-thinning blood viscosity:

(11) $$\mu =\mu_{\infty }+\left(\mu_{0}-\mu_{\infty }\right){\left[1+{\left(\lambda \dot{\gamma }\right)}^{2}\right]}^{(n-1)/2}$$

where λ, n, $\mu_{0}$ and $\mu_{\infty }$ represent the relaxation time, power-law index, zero-shear viscosity, and infinite-shear viscosity, respectively.

### 3.2. Numerical Implementation

**Figure 3:**
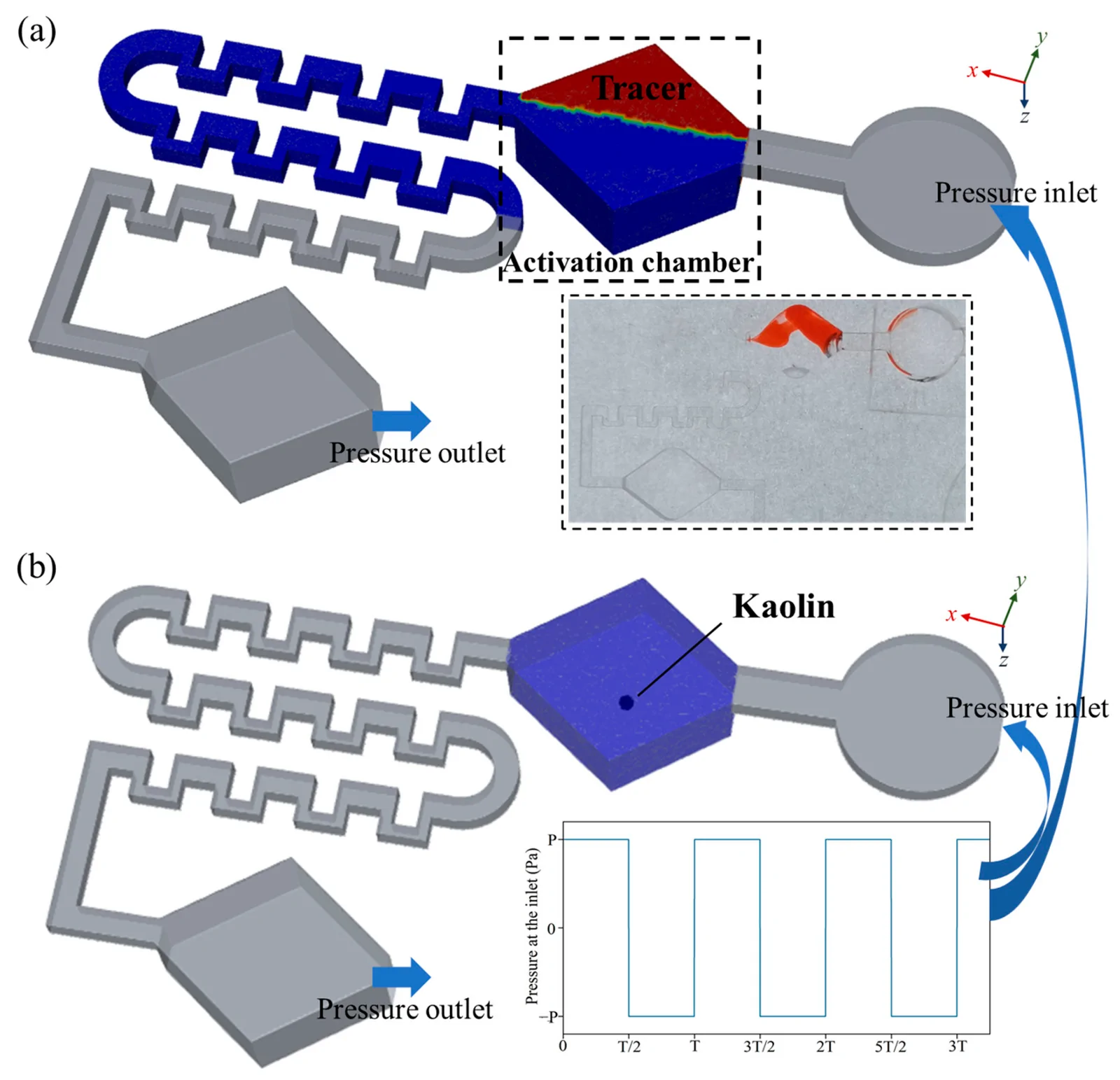
Schematic diagram of CFD model implementation. (**a**) The initialization of tracer mixing simulations referring to the experimental record. The red region is occupied by 0.5% Allura Red aqueous solution, and the blue region is full of water. (**b**) The initialization of the solid dispersion simulation with the inlet pressure condition. The blood is patched in the blue region, while Kaolin is positioned at the bottom center of the activation chamber.

To reduce the computational cost while retaining the essential flow characteristics, only the core region where the mixing and particle dispersion processes occur was extracted for numerical simulation, as shown in Figure 3. A periodic pressure boundary condition, described as a square wave function, was prescribed at the inlet of the microchannel to reproduce the reciprocating pneumatic actuation generated by repeatedly pressing and releasing the gas button. It is assumed that the forward and backward flows each occupy half of an oscillation cycle. The outlet was maintained at atmospheric pressure. The drop of pressure along the microchannel was estimated using the Hagen–Poiseuille equation, in which the additional pressure losses associated with local geometrical features, e.g., bends and sudden expansions or contractions, were neglected.

(12) $$∆P=\frac{128\mu QL}{\pi d^{4}}$$

where Q [m^3^/s] denotes the flow rate. L [m] and d [m] are the length and the equivalent radius of the microchannel, respectively.

The simulation case for tracer mixing was initialized to reproduce the experimental conditions by specifying the initial distributions of both the liquid phase and tracer species. As illustrated in Figure 3a, the initial mass fraction of the tracer in the designated region was set to 0.005, corresponding to the concentration of the pre-prepared tracer solution used in the mixing experiments. For the simulations investigating solid particle dispersion, kaolin particles were initially distributed at the bottom of the activation chamber with a volume fraction of 0.1, as shown in Figure 3b. This initial configuration was designed to represent the sedimentation of activator particles prior to oscillatory mixing, thereby enabling the evaluation of particle resuspension and dispersion under reciprocating flow. The material properties and numerical parameters adopted in all simulation cases are summarized in Table 1.

The governing equations were solved using the phase-coupled SIMPLE algorithm for pressure–velocity coupling. Spatial discretization was performed using the least-squares cell-based gradient reconstruction method, while the convective and diffusive terms were discretized using the second-order upwind scheme. For transient simulations, the bounded second-order implicit scheme was employed to ensure numerical stability and temporal accuracy. The iterative solution was considered converged when the residuals of all governing equations decreased below 1 × 10^−3^.

**Figure 4:**
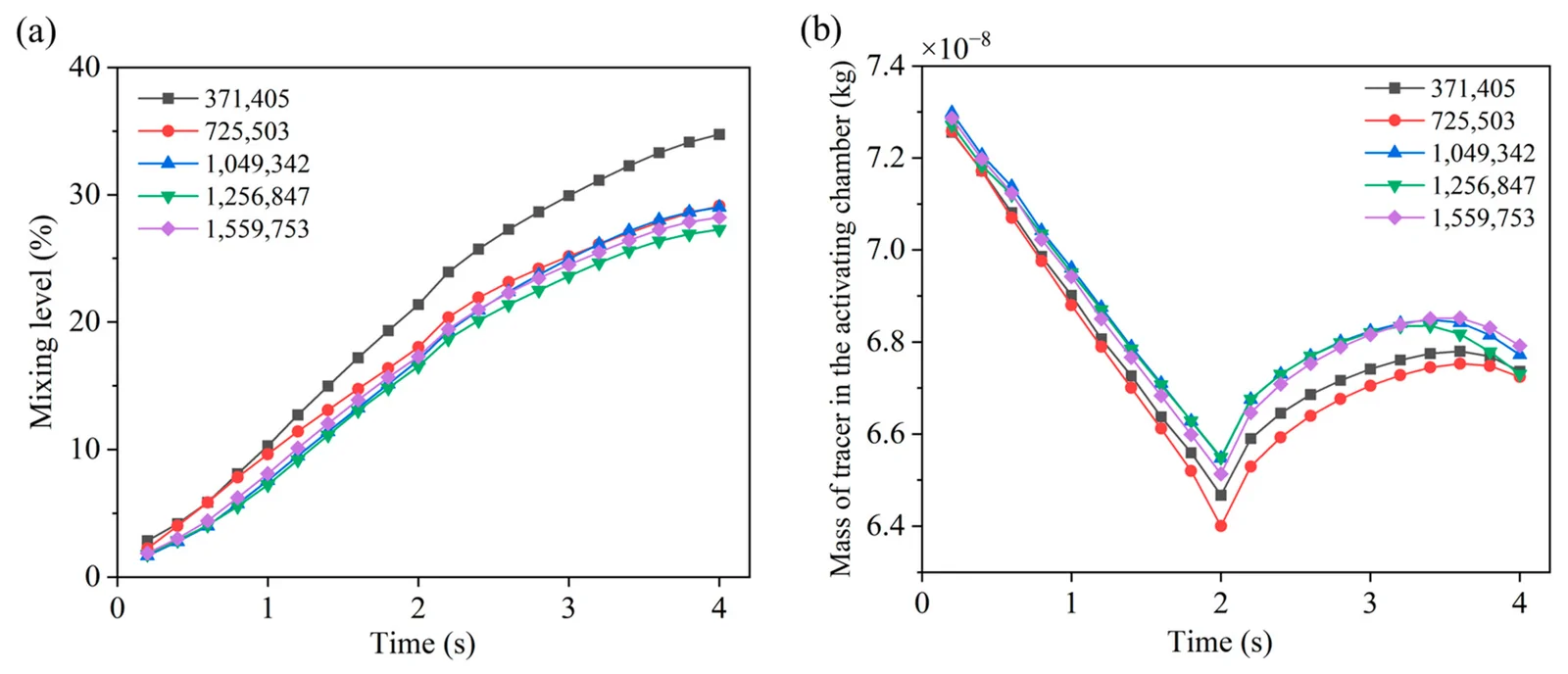
Comparison of simulation results by different grid schemes. (**a**) mixing level and (**b**) mass of tracer in the activating chamber over time.

A mesh independence study was conducted to evaluate the influence of mesh resolution on the numerical results. Five computational meshes consisting of 371,405, 725,503, 1,049,342, 1,256,847, and 1,559,753 cells were compared based on the mixing simulation case. The temporal variations in the mixing level and mass fraction of the tracer within the activation chamber obtained using the five mesh schemes are presented in Figure 4a and Figure 4b, respectively. When the number of mesh cells reached 1,049,342, the differences between successive mesh refinements became negligible. Therefore, this mesh scheme was selected for all subsequent simulations to achieve a balance between computational accuracy and efficiency.

### 3.3. Quantification of Mixing Performance

The mixing performance in the numerical simulation was evaluated on the basis of the analysis method proposed by Zou et al. [30]. The level of tracer mixing or activator dispersion at any of the grids in the 3D activation chamber can be defined by Equation (13) using their mass fraction levels. Further averaging the levels of all grids in the fluid domain by Equation (14) gives the mixing level for the entire domain.

(13) $$x=\{\begin{array}{c} \frac{m_{i,t}}{m_{\infty }}\;\;\;m_{i,t}<m_{\infty }\;\\ \frac{m_{0}-m_{i,t}}{m_{0}-m_{\infty }}\;\;m_{i,t}\geq m_{\infty } \end{array}\;$$

(14) $$M_{sim}=\frac{\sum_{i=1}^{j}\frac{m_{i,t}}{m_{\infty }}+\sum_{i=j+1}^{n}\frac{{m_{0}-m}_{i,t}}{m_{0}-m_{\infty }}}{n}\times 100\%$$

where $m_{i,t}$ is the mass fraction of the tracer or activator at grid i at moment t. $m_{0}$ is the mass fraction at the initial dyeing region. $m_{\infty }$ is the mass fraction at which the mixing region reaches fully homogeneous. n represents the number of grids in the mixing region. j is the number of the grids marked by the mass fraction below $m_{\infty }$.

### 3.4. Definition of Reynolds Number

The Reynolds number is defined by the following equation:

(15) $$Re=\frac{d_{c}\rho_{l}v_{l}}{\mu }$$

where $d_{c}$ [m] is the characteristic diameter of the activation chamber. $\rho_{l}$ [kg/m^3^] and $v_{l}$ [m/s] are density and velocity of liquid, respectively. The volume-averaged Reynolds number is used to describe the flow regime in the activation chamber:

(16) $$\hat{Re}=\frac{\int \left|Re\right|dV}{\int dV}$$

The time-averaged flow velocity and the average Reynolds number over a certain period can be calculated by:

(17) $$\bar{Re}=\frac{\int_{0}^{T}\hat{Re}dt}{T}$$

(18) $$\bar{v_{l}}=\frac{\int_{0}^{T}v_{l}dt}{T}$$

### 3.5. Analysis of Parameter Sensitivity

A sensitivity coefficient was adopted to analyze how the level of solid dispersion varies with respect to the researched parameters [31].

(19) $$S\left(\theta_{k}\right)=\frac{M\left(\theta_{k}\right)-M(\theta_{ref})}{\theta_{k}-\theta_{ref}}\times \frac{\theta_{ref}}{M(\theta_{ref})}$$

where $M\left(f_{k}\right)$ represents the level of solid dispersion corresponding to a parameter value of $\theta_{k}$. The sensitivity was normalized by the reference value $\theta_{ref}$ and its corresponding dispersion level in order to compare systems with different initial solid positions. In this work, the reference value was set to the minimum within a specific parameter range. The average sensitivity coefficient within the range was given by the following equation:

(20) $$\bar{S}=\frac{\sum_{k=1}^{N}S\left(\theta_{k}\right)}{N}$$

where N denotes the number of the researched parameter values.

## 4. Results and Discussion

### 4.1. CFD Model Validation Using the Tracer Mixing Experiment

The multi-physics CFD model of the microfluidic mixer was first validated against the tracer mixing experiment described in Section 2.2. The pressure boundary condition for the baseline simulation was estimated to be approximately 500 Pa using Equation (14), based on the geometric dimensions of the microfluidic device and the experimentally observed fluid motion. The oscillatory pressure boundary was prescribed with a period of 4 s, consisting of 2 s of positive inlet pressure followed by 2 s of negative inlet pressure.

**Figure 5:**
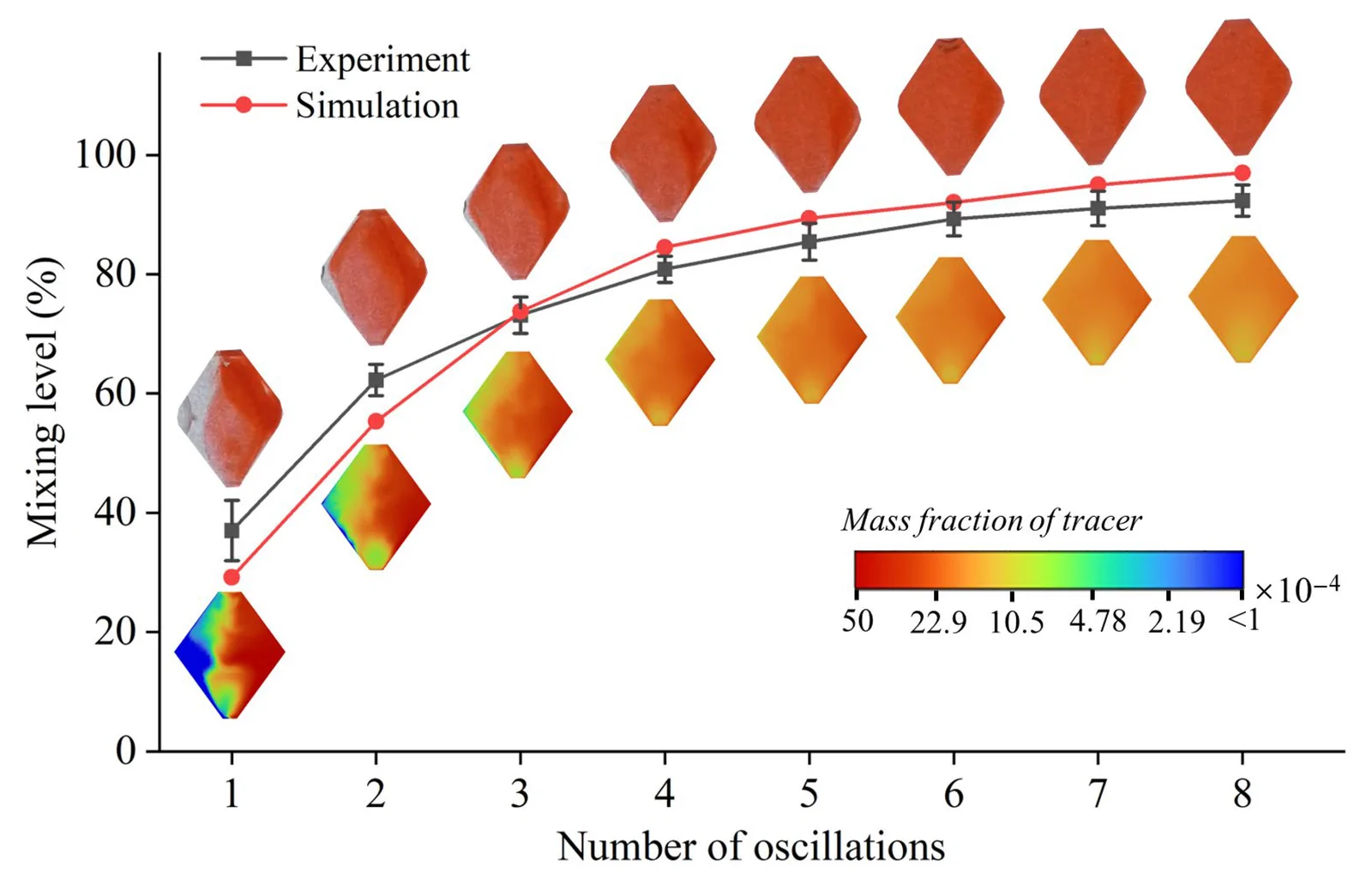
Comparison of experimental and simulation results of mixing levels in the activating chamber over time. The images above the curve are representative color photos captured by a camera. Experimental data points represent mean ± standard deviation (SD) and *n* = 3. The images below the curve are simulated tracer mass fraction contours at a height of 1 mm in the activation chamber. The color bar is displayed on a log scale.

Figure 5 compares the evolution of the mixing level in the activation chamber obtained from the experiments and numerical simulations over the number of oscillation cycles. The data were recorded after each complete oscillation cycle, corresponding to one press-and-release operation. The experimental data represent the mean values of three replicates, with error bars denoting the standard deviation. All the original color images of the reproducibility experiments are available in Table S1 of the Supplementary Materials. The first two oscillation cycles contribute most significantly to mixing enhancement, with each cycle accounting for approximately 30% of the overall increase in the mixing level. After five oscillation cycles, the color differences across the activation chamber become visually indistinguishable in the experimental images, corresponding to a mixing level of approximately 90%.

A good agreement between the experimental measurements and numerical predictions was achieved with a coefficient of determination R^2^ of 0.9266. Representative experimental images and the corresponding simulated tracer mass fraction distributions on the mid-plane of the activation chamber are also presented in Figure 5. Both the experimental observations and numerical results exhibit a consistent evolution of the mixing pattern throughout the oscillation process. This result demonstrates that the in silico model accurately captures the transient mixing behavior within the microfluidic device. The model will subsequently be employed to capture the dispersion process of activators in blood. The validation using the Newtonian glycerol solution has ensured the reliability of the CFD numerical framework, including the mesh quality, discretization schemes, and solver algorithms. Since the governing equations remain the same and only the viscosity term is updated to reflect the shear-thinning behavior of blood, the established model is capable of predicting the blood flow characteristics with high confidence.

### 4.2. Effect of Initial Loading Position on Solid Dispersion

**Figure 6:**
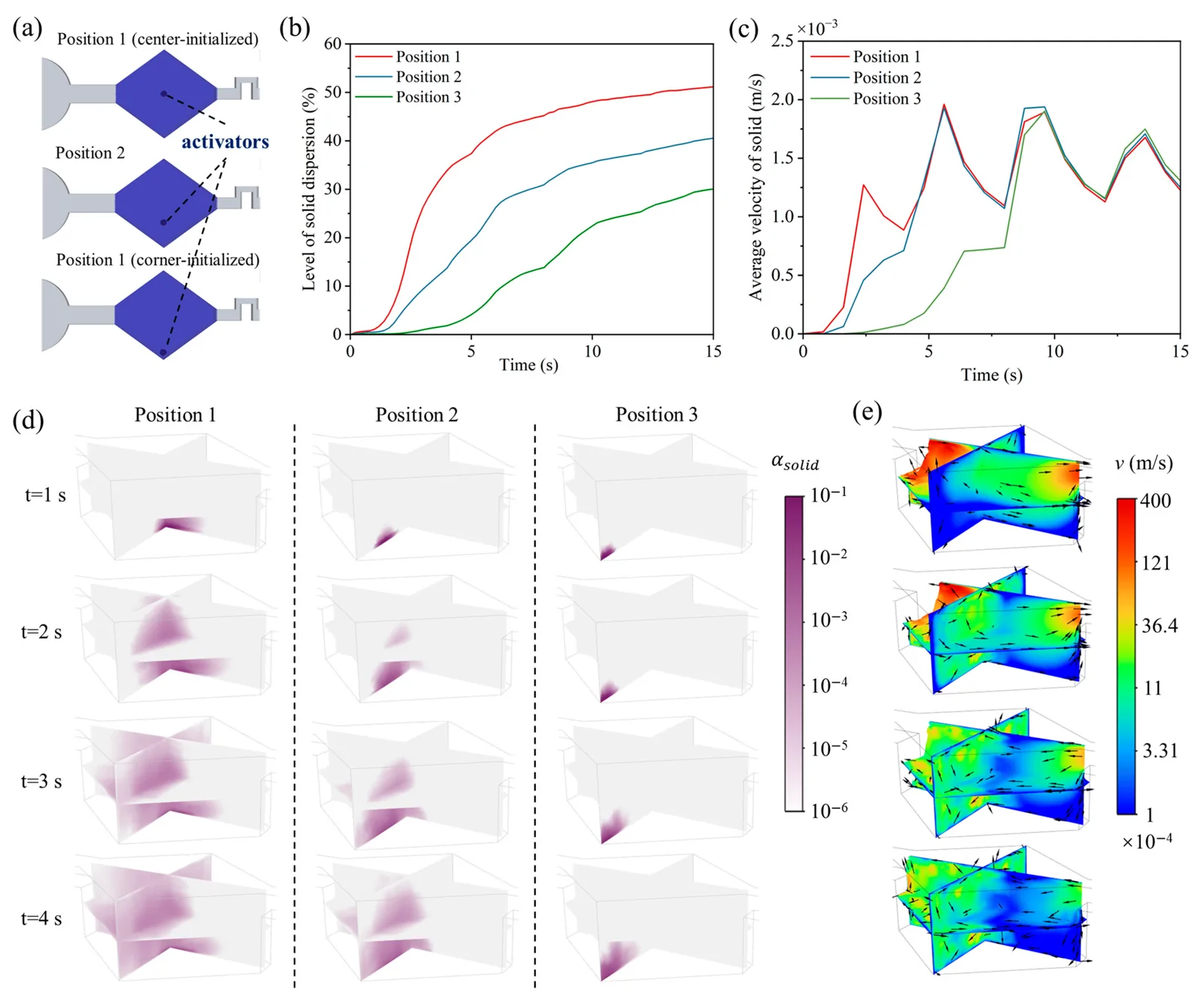
Comparison of solid dispersion for different initial solid positions. (**a**) Schematic of initial solid distribution. (**b**) Level of solid dispersion over time. (**c**) Average velocity of solid over time (**d**) Temporal evolution of solid volume distribution in the activating chamber. (**e**) Temporal evolution of flow field of liquid phase in the activating chamber. The color bars are both displayed on a log scale.

The validated CFD model was subsequently employed to investigate the mixing behavior between solid activators and blood within the micromixer. Since solid activators may settle at different locations inside the activation chamber during practical operation, the influence of the initial particle position on the dispersion process was evaluated by varying the initial packing region of the solid phase, as illustrated in Figure 6a. In all simulation cases, the kaolin particles were initially deposited on the bottom surface of the activation chamber. The material properties of blood and kaolin used in the simulations are listed in Table 1. The inlet pressure and oscillation period were maintained at 500 Pa and 4 s, respectively. Preliminary experiments utilizing Allura Red dye and glycerol solutions were conducted to qualitatively validate the numerical simulations. Detailed experimental methodologies and corresponding visualized results are presented in the Supplementary Materials.

Figure 6b shows that the initial location of the kaolin particles has a pronounced influence on the dispersion process. As the initial particle packing position shifts from the chamber center toward the corner, the solid dispersion rate decreases significantly. This trend is further confirmed by the transient particle distributions shown in Figure 6d, where particles initially located at the center of the chamber disperse much more rapidly than those deposited near the corner. At 15 s, the dispersion level of the corner-initialized case (Position 3) is 28.3% lower than that of the center-initialized case (Position 1), while the maximum difference reaches 42% at 8 s. The dispersion curves in Figure 6b further indicate that all three cases experience an initial stagnation stage before entering a period of rapid particle dispersion. Notably, this stagnation period becomes progressively longer as the initial particle position approaches the chamber corner. From Figure 6b,c, it is clear that the onset of rapid dispersion coincides with a substantial increase in the average velocity of the solid phase. During the first two oscillation cycles, the average solid velocity in Position 3 remains considerably lower than those in Positions 1 and 2. As the oscillatory flow develops, the average solid velocity in the three cases gradually converges to comparable values.

The underlying mechanism can be explained by the transient hydrodynamic characteristics shown in Figure 6e. During the pressing stage of each oscillation cycle, the flow velocity in the central region of the activation chamber is substantially higher than that near the sidewalls and corners, producing a pronounced spatial velocity gradient. According to Equation (6), a higher relative velocity between the liquid and solid phases generates a stronger interphase drag force, thereby promoting particle dispersion. The simulated flow field is consistent with those reported in previous numerical studies of microfluidic systems [32,33], exhibiting a velocity distribution that is characteristic of laminar flow. Because of the strong viscous effect within the near-wall boundary layer, the fluid velocity decreases markedly from the chamber center toward the corners. As oscillatory mixing proceeds, the repeated reciprocating motion gradually weakens this velocity gradient. Consequently, the local hydrodynamic conditions become increasingly favorable for particle resuspension and dispersion, leading to the gradual reduction in the differences observed among the three initial solid loading positions.

### 4.3. Effect of Oscillation Period on Solid Dispersion

As a key operating parameter of the proposed microfluidic mixer, the oscillation period determines both the oscillation frequency and the volume of fluid exchanged between the activation chamber and the microchannel during each reciprocating cycle. Oscillation periods of 1, 2, 3, 4, and 5 s were selected within the practical operating range for the parametric study. Two groups of simulations were performed with the particles initially deposited at the center and the corner of the activation chamber.

**Figure 7:**
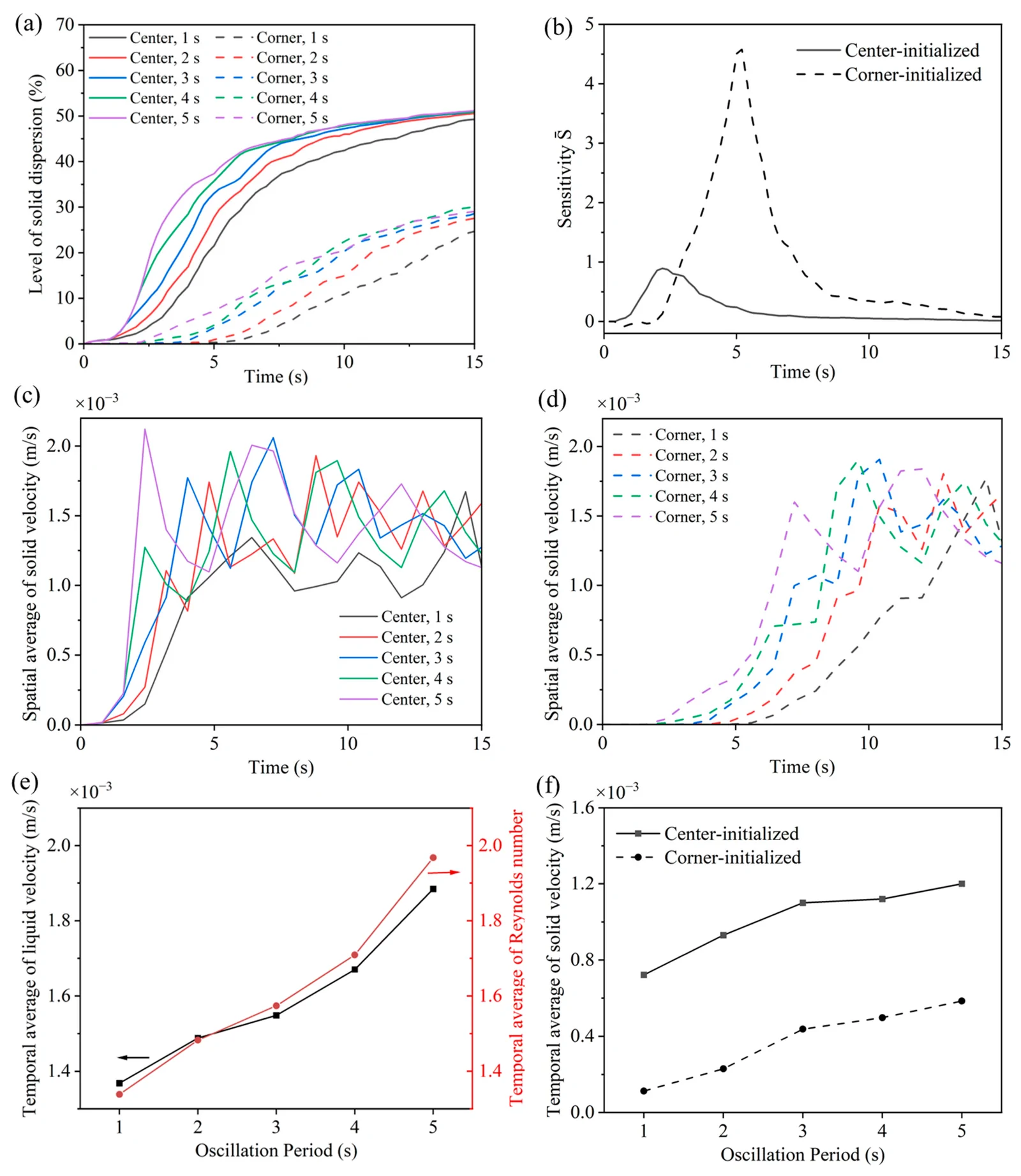
Effect of oscillation period on the solid dispersion for center-initialized and corner-initialized solid loading cases. (**a**) Evolution of the solid dispersion level. (**b**) Sensitivity of the solid dispersion level to the oscillation period. (**c**) Evolution of solid velocity for center-initialized loading cases. (**d**) Evolution of solid velocity for corner-initialized loading cases. (**e**) Temporal averages of liquid velocity and Reynolds number over the first 10 s. (**f**) Temporal average of solid velocity over the first 10 s.

**Figure 8:**
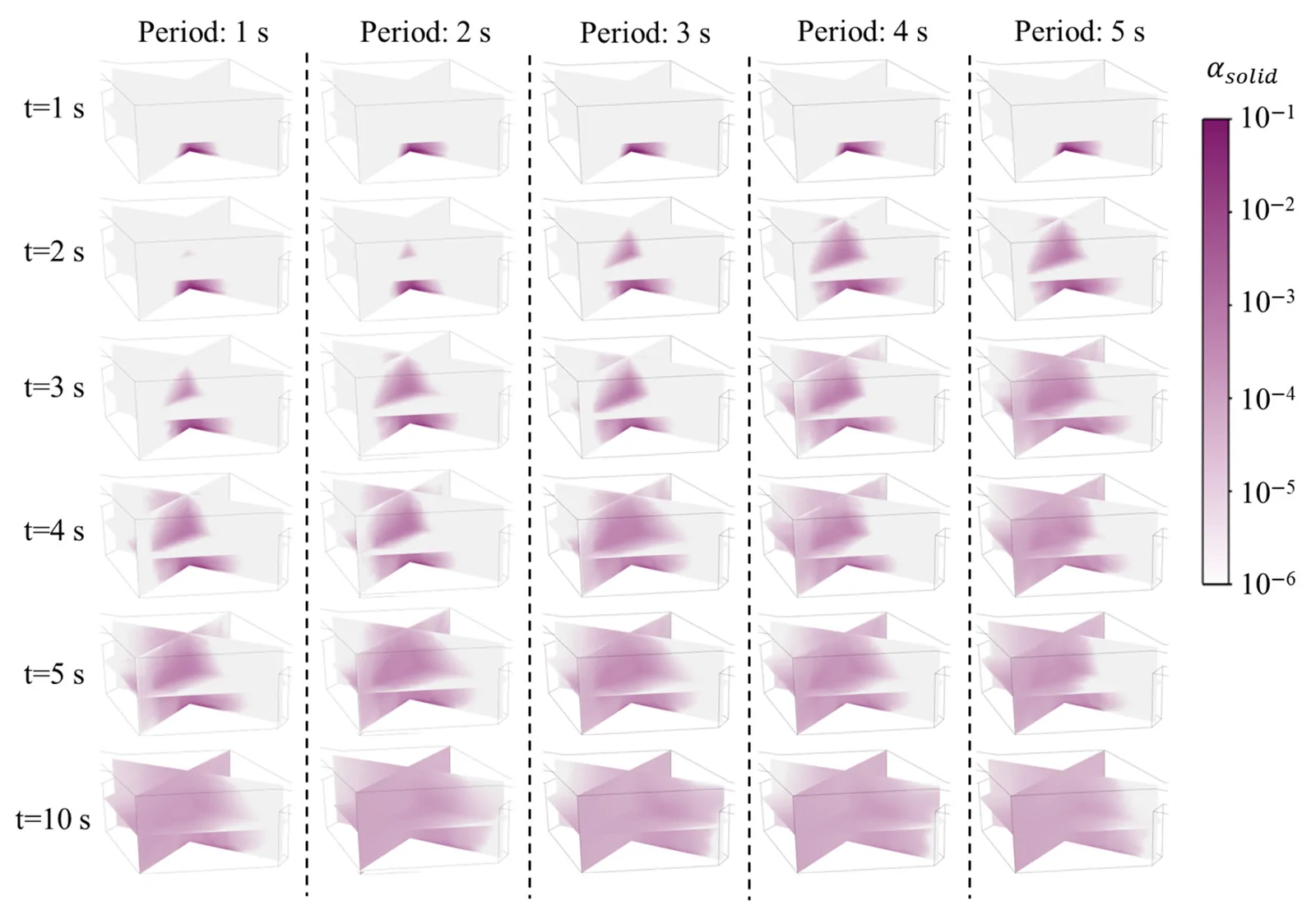
Evolution of solid volume fraction when the solid is initially located at the center of the activation chamber.

**Figure 9:**
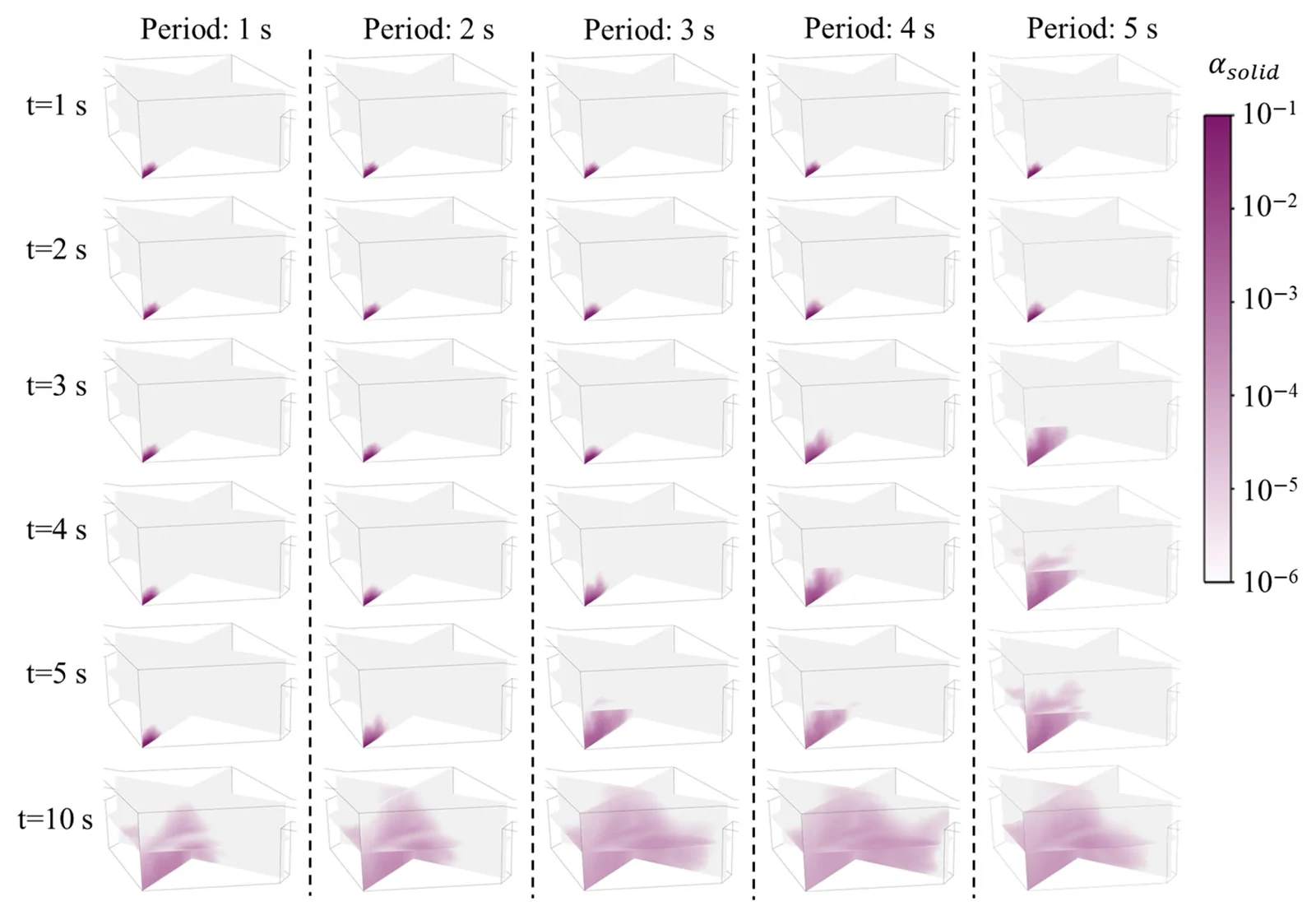
Evolution of solid volume fraction when the solid is initially located at the corner of the activation chamber.

The influence of the oscillation period on the solid dispersion level for both initial solid configurations is presented in Figure 7a. Increasing the oscillation period consistently enhances the solid dispersion performance. This trend is further confirmed by the transient particle distributions shown in Figure 8 and Figure 9, where longer oscillation periods lead to accelerated solid dispersion. Moreover, Figure 7b shows that the influence of the oscillation period is considerably more pronounced when the Kaolin particles are initially deposited near the chamber corner, indicating that oscillatory conditions play a greater role in overcoming the unfavorable hydrodynamic environment in this region.

The evolution of the spatially averaged particle velocity shown in Figure 7c,d provides further insight into this behavior. Although the particle velocities under different oscillation periods gradually converge during the later stage of mixing, longer oscillation periods produce a faster increase in particle velocity during the initial dispersion stage, resulting in a higher time-averaged particle velocity throughout the process, as given in Figure 7f. Correspondingly, the divergence of the dispersion curves in Figure 7a also develops during this velocity growth stage. The sensitivity analysis in Figure 7b further demonstrates that the influence of the oscillation period is mainly confined to the first 5 s for the center-initialized cases, whereas it persists from approximately 2 to 15 s for the corner-initialized cases.

A closer examination of Figure 7c,d reveals that the particle velocity decreases during the backflow stage of every oscillation cycle. In addition, the differences among the various oscillation periods first become apparent at the onset of the initial backflow. It implies that oscillatory motion periodically disturbs the laminar flow and weakens the near-wall velocity gradient, but the reverse flow simultaneously reduces the net momentum in the activation chamber. Consequently, shorter oscillation periods introduce more frequent backflows, thereby limiting the continuous acceleration of the solid. This interpretation is further supported by Figure 7e, which shows that increasing the oscillation period increases the average liquid velocity within the activation chamber during the first 10 s. It is noteworthy that the corresponding time-averaged Reynolds number increases to a greater extent. As blood exhibits shear-thinning behavior, the higher shear rate generated under a longer oscillation period reduces the apparent viscosity predicted by the Carreau model, thereby further increasing the Reynolds number.

The numerical observations are consistent with previous experimental studies on oscillatory flow reactors. For example, using a continuous oscillatory tubular reactor (2 m in length and 26 mm in width), Slavnić et al. [34] reported that increasing the oscillation frequency (i.e., decreasing the oscillation period) reduced the axial dispersion of millimeter-sized particles. Similarly, Kramer et al. [35] investigated the residence time distributions of homogeneous and heterogeneous tracer systems and found that lower oscillation amplitudes resulted in poorer solid dispersion. Their finding can also match the simulation result in the present study. When the inlet pressure is controlled to be constant, a longer pressurization duration results in a longer oscillation period and consequently a larger fluid displacement.

### 4.4. Biparametric Orthogonal Analysis

Following the investigation of the individual operating parameters, their combined effects on solid dispersion were further analyzed using response surface regression. For quantitative comparison, the initial position of the solid activator was represented by the normalized distance from the center of the activation chamber. A quadratic polynomial regression model was employed to establish the quantitative relationship between the simulated parameters and the predicted results. The coefficient of determination R^2^ was used to evaluate the fitting effect of the response surface regression. A value closer to 1 indicates a better fit.

**Figure 10:**
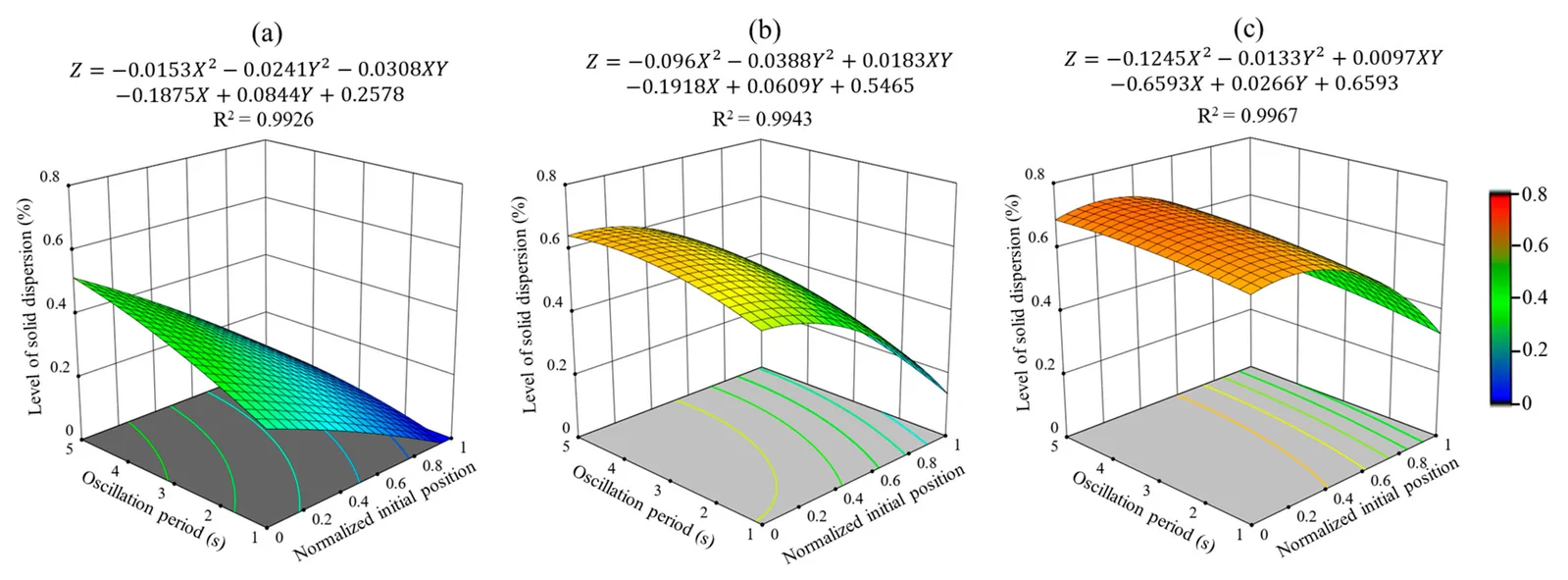
Response surface plots of solid dispersion level against oscillation period and initial loading position at (**a**) 5 s, (**b**) 10 s and (**c**) 15 s. *X*: normalized initial loading position; *Y*: oscillation period; *Z*: solid dispersion level.

Figure 10a–c present the response surfaces describing the combined influence of the initial particle position and oscillation period on the solid dispersion level at different times, respectively. Additionally, the regression models corresponding to the response surfaces are presented above the figures, demonstrating high R^2^ values. At 5 s, both the initial particle position and the oscillation period exert a pronounced influence on the dispersion performance, as evidenced by the steep gradients of the response surface. As the mixing process proceeds to 10 s, the effect of the oscillation period remains significant when the particles are initially deposited near the chamber corner, whereas its influence gradually diminishes for particles initially located closer to the chamber center. By 15 s, the response surface becomes nearly independent of the oscillation period at a given initial particle position.

The response surface analysis demonstrates that the sensitivity of solid dispersion to the investigated operating parameters is strongly time-dependent. During the early stage, both the initial solid loading position and the oscillation period jointly govern particle suspension and transport. As the oscillatory flow develops, the influence of the oscillation period gradually weakens, while the initial solid position remains the dominant factor determining the overall dispersion efficiency. These findings can provide useful guidance for selecting appropriate operating conditions according to the required mixing time in practical ACT assays.

## 5. Conclusions

In this study, a multi-physics CFD model was developed to investigate the solid dispersion behavior in a novel oscillatory flow micromixer designed for rapid activation of the coagulation cascade. The reliability of the numerical model was successfully validated against tracer mixing experiments. The established model enabled detailed characterization of the microscale hydrodynamic behavior and solid–liquid dispersion process under different operating conditions, which are difficult to quantify experimentally.

Sample calculations demonstrate that the initial loading position of kaolin particles has a significant influence on their dispersion efficiency in blood. Particles initially deposited at the chamber corner exhibit slower dispersion due to the reduced flow velocity in the near-wall region. Increasing the oscillation period can enhance solid dispersion by extending the forward transport duration of the liquid phase, although the improvement strongly depends on the initial particle position. By resolving the transient solid–liquid interaction, the CFD analysis further reveals that the reverse-flow stage reduces the liquid velocity and consequently weakens the drag force acting on the particles. Therefore, compared with continuous unidirectional flow, oscillatory flow does not directly improve particle dispersion through sustained momentum transfer. Instead, its primary advantage lies in substantially prolonging the residence time of a small liquid volume within the mixing region and repeatedly disturbing the local flow field.

While this micromixer has been applied in ACT testing in our recent publications [36,37], the present findings indicate that rapid and homogeneous mixing between blood and solid activators requires both optimized activator placement and appropriate oscillatory operating conditions. However, accurately controlling the initial particle loading location remains challenging due to the microscale dimensions of the activation chamber. Future efforts will focus on optimizing the chamber geometry to improve near-wall flow characteristics. Another critical challenge for this micromixer is that the oscillatory flow induces the generation of bubbles in the blood, which is detrimental to coagulation assays. In addition, it should be noted that the oscillatory actuation employed in this micromixer inevitably induces air-liquid interface instability, which may lead to bubble formation within the blood sample. The bubble-liquid interface can activate the intrinsic coagulation pathway independently of the solid activator or even lead to hemolysis, thereby confounding the assay results. Further CFD-based parametric analysis is required to balance solid dispersion performance and bubble suppression for reliable coagulation assays.

## Figures and Tables

**Table 1:** Parameter values used in the simulation.

Parameters	Value	Reference
Density of 20% glycerol aqueous solution	1050 kg/m^3^	Measured
Viscosity of 20% glycerol aqueous solution	2.6 × 10^−3^ Pa·s	Measured
Molecular Weight of Allura Red	496 g/mol	[26]
Diffusivity of Allura Red	2.1 × 10^−10^ m/s^2^	Estimated
Initial mass fraction of Allura Red	0.5%	[-]
Density of whole blood	1050 kg/m^3^	[27]
Density of Kaolin	2600 kg/m^3^	[28]
Particle size of Kaolin	2 μm	[29]
Total mass of Kaolin	1.6 μg	[-]
Initial packing volume fraction of Kaolin	0.1	[-]
Relax time of Carreau model	3.313 s	[25]
Power-law index of Carreau model	0.3568	
Zero-shear viscosity of Carreau model	0.056 Pa·s	
Infinite-shear viscosity of Carreau model	0.0035 Pa·s	

## Data Availability

The data presented in this article are available on request from the corresponding authors.

## Supplementary Materials

The following supporting information can be downloaded at: https://www.mdpi.com/article/10.3390/mi17091036/s1, Table S1: Color images of the activation chamber from the reproducibility experiments; Table S2: Comparison of experimental results between the solid center-loading and solid corner-loading schemes.
