# Supplementary material for: Solid Dispersion Characteristics of an Oscillatory Microfluidic Mixer: An Experimental and Numerical Investigation
Source: Micromachines (Basel). 2026 Aug 29;17(9):1036. doi: 10.3390/mi17091036 (PMC13609804; doi:10.3390/mi17091036)
Supplement: Supplementary file 1 [file micromachines-17-01036-s001.zip › micromachines-4503623-supplementary.pdf]

Supplementary Materials

Solid Dispersion Characteristics of an Oscillatory Microfluidic Mixer: An Experimental and Numerical Investigation

Yao Lu [1,2], Haixuan Sun [1,2,*] and Yifan Qin [1,*]

[1] Suzhou Institute of Biomedical Engineering and Technology, Chinese Academy of Sciences, Suzhou 215163, China; luyao@sibet.ac.cn
[2] School of Biomedical Engineering (Suzhou), University of Science and Technology of China, Hefei 230026, China
* Correspondence: sunhx@sibet.ac.cn (H.S.); qinyf@sibet.ac.cn (Y.Q.)

Table S1 shows all the original color images of the reproducibility experiments for CFD model validation.

Table S1 Color images of the activation chamber from the reproducibility experiments.

Group order	Oscillation number									
	0	1	2	3	4	5	6	7	8	∞
1	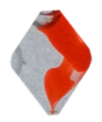	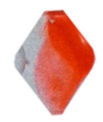	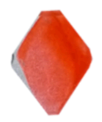	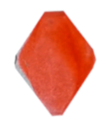	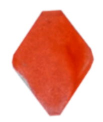	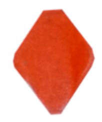	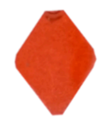	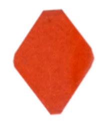	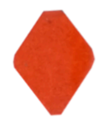	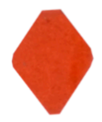
2	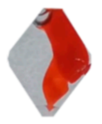	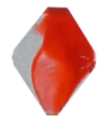	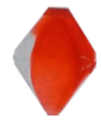	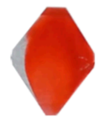	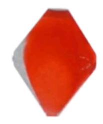	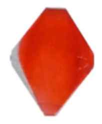	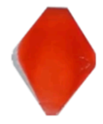	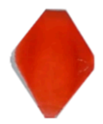	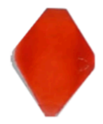	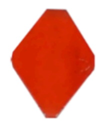
3	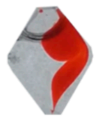	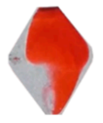	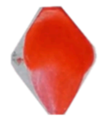	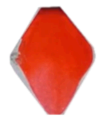	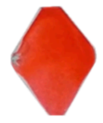	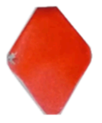	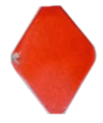	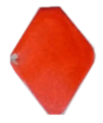	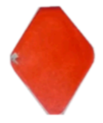	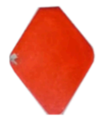

Two additional experiments were conducted to verify the simulation result regarding the effect of initial loading position on solid dispersion. A 1.5 μL droplet of 0.5% Allura Red solution was dispensed into the center and the corner of the activation chamber, respectively. Lyophilization (Laboratory Freeze Dryer, Tofflon, Shanghai, China) was then employed to obtain the initial solid loading configurations shown in Table S2. The pneumatic unit was operated to induce oscillatory flow of a 30 μL of 20 % glycerol solution within the activation chamber and microchannel. A high-definition camera was then used to capture the color changes in the activation chamber.

It was observed that the tracer loaded in the center dispersed throughout the entire chamber more rapidly as oscillation progressed. In contrast, the corner-loaded case retained a deeper red region in the corner, indicating poorer solid dispersion efficiency. The experimental results qualitatively demonstrate the significant effect of the initial position of the solid activator on the dispersion performance of the micromixer, which is consistent with the simulation results (Figure 8 and Figure 9).

Table S2 Comparison of experimental results between the solid center-loading and solid corner-loading schemes.

Solid loading position	Oscillation number								
	0	1	2	3	4	5	6	7	8
Center	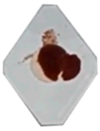	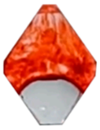	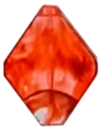	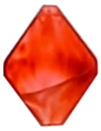	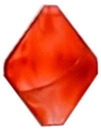	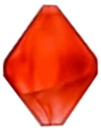	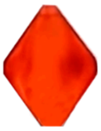	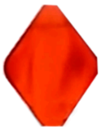	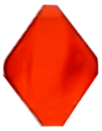
Corner	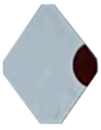	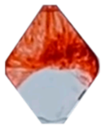	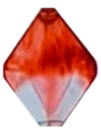	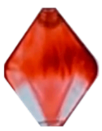	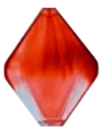	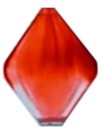	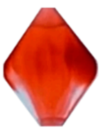	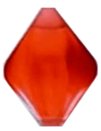	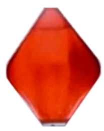
